# Extrakorporale Verfahren in der Lungentransplantation und darüber hinaus

**DOI:** 10.1055/a-2563-8383

**Published:** 2025-04-07

**Authors:** Alexander Hermann, Thomas Schweiger

**Affiliations:** 1 27271 Universitätsklinik für Innere Medizin I, Medizinische Universität Wien, Wien, Österreich; 2 27271 Universitätsklinik für Thoraxchirurgie, Medizinische Universität Wien, Wien, Österreich

**Keywords:** Lungentransplantation, extrakorporale Membranoxygenierung, Atemwegschirurgie, lung transplantation, extracorporale membrane oxygenation, airway surgery

## Abstract

Die Lungentransplantation stellt bei Patient*innen mit Lungenerkrankungen im Endstadium oft die einzige und letzte Behandlungsoption dar. In den letzten Jahren konnten die Ergebnisse nach Lungentransplantation stetig verbessert werden, nicht zuletzt wegen der technischen Entwicklungen im Bereich der Lungenersatzverfahren. Extrakorporale Gasaustauschverfahren sind heute integraler Bestandteil des klinischen Alltags und insbesondere an Lungentransplantationszentren. Der Ersatz reicht von relativ einfachen, pumpenlosen Membranen bis hin zu aufwendigen Kanülierungsarten und Gerätekonfigurationen. Insbesondere bei der extrakorporalen Membranoxygenierung (ECMO) unterscheidet man eine Reihe von Konfigurationen, die in der Thoraxchirurgie und speziell bei der Lungentransplantation Anwendung finden. Des Weiteren kann man hier zwischen der präoperativen ECMO, dem sog. Bridge-to-Transplant, sowie der intraoperativen ECMO-Unterstützung während der Transplantation unterscheiden. Diese hat in den letzten Jahren die Herz-Lungen-Maschine weitgehend verdrängt. Auch unmittelbar postoperativ spielt die ECMO bei Verhinderung und Therapie der primären Graft-Dysfunktion eine entscheidende Rolle. Neben der Lungentransplantation finden die extrakorporalen Unterstützungssysteme auch bei erweiterten thoraxchirurgischen Resektionen oder Eingriffen an den zentralen Atemwegen Anwendung. Auch zukünftig werden extrakorporale Verfahren durch die technischen Weiterentwicklungen und Verbesserungen in den Behandlungsprotokollen eine Schlüsselrolle in der Versorgung thoraxchirurgischer Patient*innen spielen.

## Abstract (en)

Lung transplantation is often the sole and final therapeutic option for patients with end-stage lung disease. In recent years, clinical outcomes have steadily improved, driven in part by advancements in extracorporeal mechanical support. These techniques have become an integral component of routine clinical practice, particularly in lung transplantation centres. Extracorporeal lung support systems range from relatively simple pumpless devices to complex cannulation techniques and device configurations. In particular, extracorporeal membrane oxygenation (ECMO) encompasses various configurations widely utilised in thoracic surgery, especially in lung transplantation. A distinction can be made between preoperative ECMO, referred to as bridge-to-transplant, and intraoperative ECMO support, which is employed during the transplantation procedure. In recent years, intraoperative ECMO has largely replaced the traditional heart-lung machine. Additionally, ECMO plays a critical role in the immediate postoperative period, both in preventing and managing primary graft dysfunction. Beyond lung transplantation, extracorporeal life support systems are also applied in complex thoracic surgical resections and procedures involving the central airways. In the future, technical advancements and improvements in treatment protocols are expected to further enhance the role of extracorporeal mechanical support in the management of thoracic surgical patients.

## Einleitung

Extrakorporale Verfahren, insbesondere die extrakorporale Membranoxygenierung (ECMO), haben in verschiedensten Bereichen der Medizin Einzug gehalten. Die Technologie bietet je nach Anwendung einen temporären Ersatz des pulmonalen Gasaustausches sowie eine hämodynamische Stabilisierung der Patient*innen. Anfänglich mit überschaubaren Ergebnissen eingesetzt, hat sich das Anwendungsspektrum des extrakorporalen Lungenersatzes in den letzten Jahrzehnten erheblich erweitert, auch dank technischer Fortschritte wie optimierten Oxygenatoren, heparinbeschichteten Schlauchsystemen und verbesserten Pumpen 1 .

Im Kontext der Lungentransplantation spielt die ECMO prä-, intra- und postoperativ eine zunehmend zentrale Rolle 2 . Beim „Bridging“ zur Transplantation ist die ECMO oft die einzige Möglichkeit, Patient*innen zu stabilisieren, bis ein geeignetes Spenderorgan zur Verfügung steht. Idealerweise werden Patient*innen unter laufender ECMO-Unterstützung wach geführt und mobilisiert. Intraoperativ hat die ECMO die Herz-Lungen-Maschine (HLM) weitgehend ersetzt und ermöglicht eine schonendere und dennoch sichere Transplantation, selbst bei komplexen Patient*innen. Auch postoperativ ist die ECMO essenziell, um bei initial schlechter Graft-Funktion oder kardiorespiratorischer Instabilität die Patient*innen zu stabilisieren und dennoch lungenprotektive Strategien zu verfolgen. Auch in anderen Aspekten der Thoraxchirurgie, wie erweiterten Resektionen und Atemwegseingriffen, hat die ECMO einen festen Stellenwert und findet breite Anwendung.

Nachfolgend werden grundlegende ECMO-Konfigurationen und deren Anwendungen im Kontext der Lungentransplantation sowie der allgemeinen Thoraxchirurgie beleuchtet.

## Konfigurationsarten

### Venovenöse ECMO

**Abb. 1:**
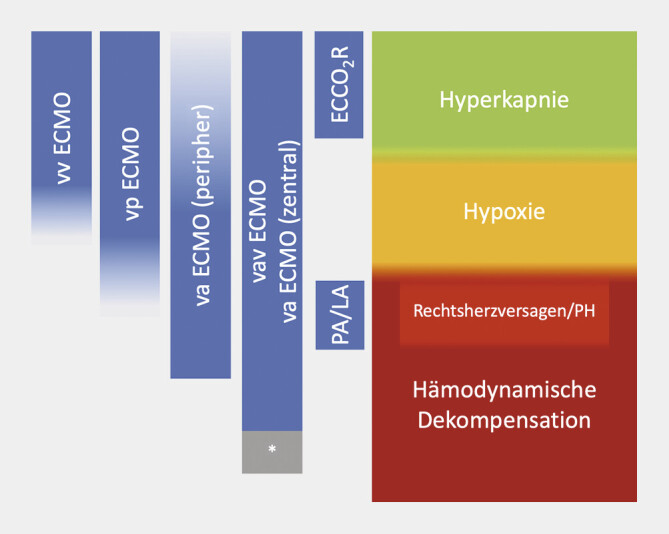
Verschiedene ECMO-Konfigurationen und deren Anwendung. Die ECMO kann in speziellen Situationen auch um zusätzliche Verfahren (*), wie etwa eine Impella erweitert werden.

Die venovenöse (VV) ECMO ist das Standardverfahren im respiratorischen Versagen ( Abb. 1 ). Mit entsprechend dimensionierten Kanülen kann ein vollständiger Lungenersatz erreicht werden. Während Blutflussraten bis 1,5 l/min für eine effektive Decarboxylierung i. d. R. ausreichen, sind für einen zusätzlichen Oxygenierungseffekt höhere Blutflüsse notwendig 3 . Systeme zur reinen Decarboxylierung (sog. Extracorporeal CO _2_ -Removal – ECCO _2_ R) finden aufgrund der limitierten Blutflussrate, insbesondere im Kontext der Lungentransplantation, mittlerweile wenig Anwendung, obwohl der Einsatz sog. „Higher-Extraction Devices“ (Blutfluss > 0,5 l/min) zum Bridging bei primär hyperkapnischem Lungenversagen teils vielversprechende Erfolge erbringen konnte 4 .

Die Standardkonfiguration der VV ECMO in den meisten Zentren ist eine femorale Drainagekanüle und eine juguläre rückführende Kanüle, wenn auch vereinzelt die jugulofemorale Betriebsart gewählt wird. Diese werden typischerweise in Seldinger-Technik eingebracht. Die Verwendung des Ultraschalls vor, während und nach der Kanülierung ist mittlerweile als Standard anzuerkennen. Bei fehlenden Kanülierungsmöglichkeiten der oberen Körperhälfte findet eine femorofemorale VV ECMO Anwendung. Hier werden 2 unterschiedlich lange und verschieden designte Kanülen über die linke und rechte V. femoralis eingebracht. Die kürzere, distal gelegene Kanüle dient zur Drainage, wohingegen die längere, rückführende Kanüle weiter zentral in der V. cava inferior zu liegen kommt. Wie auch bei der femorojugulären VV ECMO sollte zwischen den Kanülenspitzen zumindest 5–10 cm Abstand eingehalten werden, um eine Rezirkulation zwischen den Kanülen zu vermeiden.

**Abb. 2:**
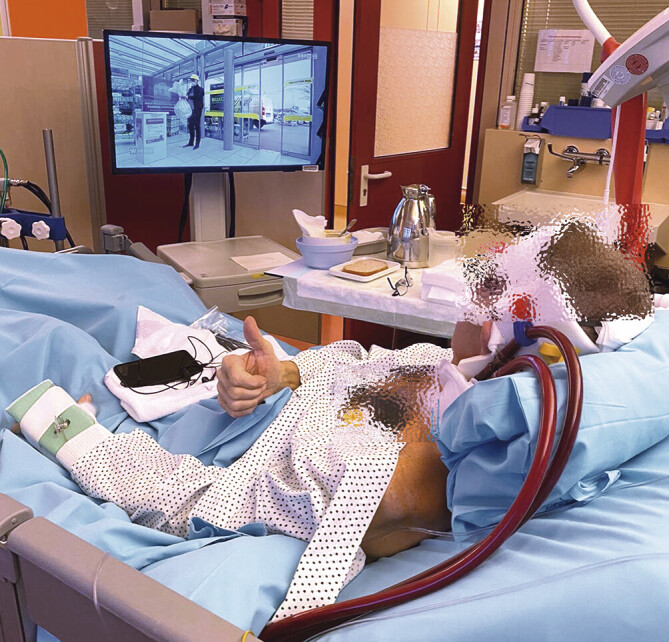
In einem wachen Bridge-to-transplant-Konzept, hier mit einer über die linke V. jugularis eingebrachten Doppellumenkanüle, kann eine Dekonditionierung durch Physiotherapie und durch aktive Interaktion mit dem Umfeld weitgehend verhindert werden.

Eine Sonderform der VV ECMO-Kanülierung stellen die Doppellumenkanülen dar ( Abb. 2 ). Durch 2 Lumina innerhalb einer Kanüle mit entsprechenden Ansaug- und Auslassöffnungen kann eine VV ECMO-Unterstützung mit lediglich einer Kanüle durchgeführt werden. Zur Kanülierung wird typischerweise die rechte V. jugularis verwendet und die Kanülenspitze bis in die V. cava inferior eingeführt. Hierzu bedarf es entsprechender Bildgebung, um ein akzidentielles Vorschieben in den rechten Ventrikel auszuschließen und die korrekte Positionierung zu ermöglichen. Zur Visualisierung sind transthorakaler Ultraschall, transösophagealer Ultraschall, Durchleuchtung oder repetitive Röntgenaufnahmen je nach Institution und Verfügbarkeit geeignet 5 . Da nur eine einzige ausreichend große Vene zugänglich sein muss, eignet sich diese Kanülierungsart besonders für Patient*innen mit sehr limitierten Zugangsmöglichkeiten. Kürzlich wurde die bisher größte Serie mit „atypischen“ Kanülierungslokalisationen für Doppellumenkanülen (d. h. V. subclavia, V. jugularis links, V. femoralis) publiziert, wobei es zu keinen kanülierungsassoziierten Komplikationen kam 6 .

Eine Limitierung der VV ECMO, insbesondere bei ausgeprägten Lungenerkrankungen, ist die Rechtsherzbelastung und pulmonale Hypertension. Diese kann bereits vorbestehen oder sich oft auch im Verlauf der VV ECMO-Therapie deutlicher ausprägen. Mehrere ECMO-Strategien kommen hier als mögliche Lösung infrage. Einerseits kann durch den Decarboxylierungseffekt eines VV Systems bereits eine gewisse Senkung des pulmonalarteriellen Drucks erwartet werden 7 . Um den Lungenkreislauf und das Rechtsherz jedoch aktiv zu entlasten, ist eine venoarterielle (VA) oder venoarteriovenöse (VAV) ECMO zu etablieren bzw. ein laufendes System entsprechend umzukonfigurieren; auf diese wird in den nachfolgenden Abschnitten eingegangen. Andere Möglichkeiten sind das interventionelle Setzen eines Atriumseptumdefekts, um einen Rechts-links-Shunt zu erzeugen 8 . Eine ECMO-Variante mit Rechtsherzunterstützung stellt auch die Kanülierung mittels Protek-Duo (CardiacAssist Inc., Pittsburgh, PA, USA) dar. Diese Doppellumenkanüle wird über die V. jugularis bis in den Hauptstamm der Pulmonalarterie eingebracht, wird daher als venopulmonale (VP) ECMO bezeichnet und erlaubt dadurch eine Rechtsherzunterstützung 9 . Aufgrund der Komplexität beider Verfahren bzw. der erst sehr rezenten Beschreibung sind diese bisher weniger verbreitet als die einfacher durchzuführende VA ECMO.

### Venoarterielle ECMO

Die VA ECMO bietet hämodynamische Unterstützung prinzipiell für Patient*innen mit oder ohne Lungenversagen. Verschiedene periphere und zentrale Kanülierungsarten sind etabliert und finden je nach Situation Anwendung. Peripher wird meist femorofemoral in Seldinger-Technik oder in teilchirurgischer Technik (sog. „Cut-down“) kanüliert. Aus der V. cava inferior wird Blut entnommen und anschließend über die rückführende Kanüle in die Aorta retrograd re-infundiert. Nachteil der peripheren VA ECMO liegen zum einen in kanülierungsassoziierten Komplikationen, wie z. B. Thromboembolien, Gefäßverletzungen und -verschlüssen, Blutungen und Wundinfektionen. Zum anderen kann es insbesondere bei Patient*innen mit respiratorischem Versagen, aber vorhandener linksventrikulärer Funktion zum sog. Harlequin-Phänomen kommen. Am Ort des Zusammentreffens beider Blutflüsse (d. h. des retrograden ECMO-Blutflusses einerseits und des genuinen kardialen Auswurfes andererseits) entsteht ein Umschlagpunkt in Form einer Wasserscheide. Je nach deren Lokalisation werden manche aortalen Gefäßabgänge entweder mit gut gasausgetauschtem ECMO-Blut oder andere mit unzureichend oxygeniertem Blut aus dem linken Ventrikel versorgt. Diese Wasserscheide zwischen gut oxygeniertem und weniger oxygeniertem Blut kann sich durch verschiedene Einflussfaktoren verschieben und muss daher rechtzeitig erkannt werden. Als Grundregel sollte bei Patient*innen mit VA ECMO daher die Pulsoxymetrie sowie die arterielle Blutentnahme am rechten Arm erfolgen. Hirnsättigungsmonitoring, z. B. mittels Nahinfrarotspektroskopie (NIRS), kann unter Kenntnis ihrer Limitationen ebenso zusätzliche Hinweise auf ungleiche Durchblutung liefern wie kontrastmittelgestützter Gefäßultraschall 10 11 .

Zusätzlich wird meist eine antegrade, kleinlumige arterielle Kanüle in die A. femoralis eingebracht, um eine Beinischämie zu vermeiden. Ein pulsoxymetrisches und klinisches Monitoring der Beinreperfusion ist ebenfalls obligat.

Bei zentraler VA ECMO kann ein Harlequin-Phänomen vermieden werden, da der rückführende Schenkel der ECMO in die Aorta ascendens eingebracht wird. Nachteil ist wiederum der chirurgische Zugang, wobei Thorakotomie, Sternotomie oder Clamshell-Inzision allesamt geeignet sind, um eine zentrale VA ECMO zu etablieren. Eine Sonderform stellt die sog. „Sport“-Konfiguration dar. Hierbei wird z. B. aus der V. jugularis venöses Blut drainiert und über einen Graft an der A. subclavia das oxygenierte Blut rückgeführt. Neben spezifischen Komplikationen wie die des Hyperperfusionssyndroms des Armes hat diese Konfiguration allerdings Vorteile im Hinblick auf die Mobilisierbarkeit der Patient*innen 12 .

### Venoarteriovenöse ECMO

Die Notwendigkeit einer VAV ECMO ergibt sich vor allem zum einen bei einer rechtskardialen Dekompensation unter laufender VV ECMO oder bei refraktärem Harlequin-Phänomen und bereits etablierter VA ECMO. Bei der VAV ECMO wird venöses Blut entnommen und zum einen in eine andere Vene und zusätzlich in eine Arterie abgegeben. Die meistverwendete Konfiguration ist eine femorale Drainagekanüle über die V. femoralis und je eine rückführende Kanüle über die V. jugularis und A. femoralis. Der Rückfluss wird über ein Y-Stück geteilt. Beide Schenkel müssen kontinuierlich mittels Flusssensoren gemonitort werden, da der jeweilige Fluss Schwankungen unterliegt und von verschiedensten Faktoren abhängt. Über Schlauchklemmen kann der Widerstand in den Schenkeln behelfsmäßig reguliert und so die Flussmenge bis zu einem gewissen Grad gesteuert werden. Auch mithilfe einer Doppellumenkanüle kann durch das zusätzliche Einbringen einer arteriellen Kanüle eine VAV ECMO etabliert werden 13 .

### Weitere Konfigurationen

**Abb. 3:**
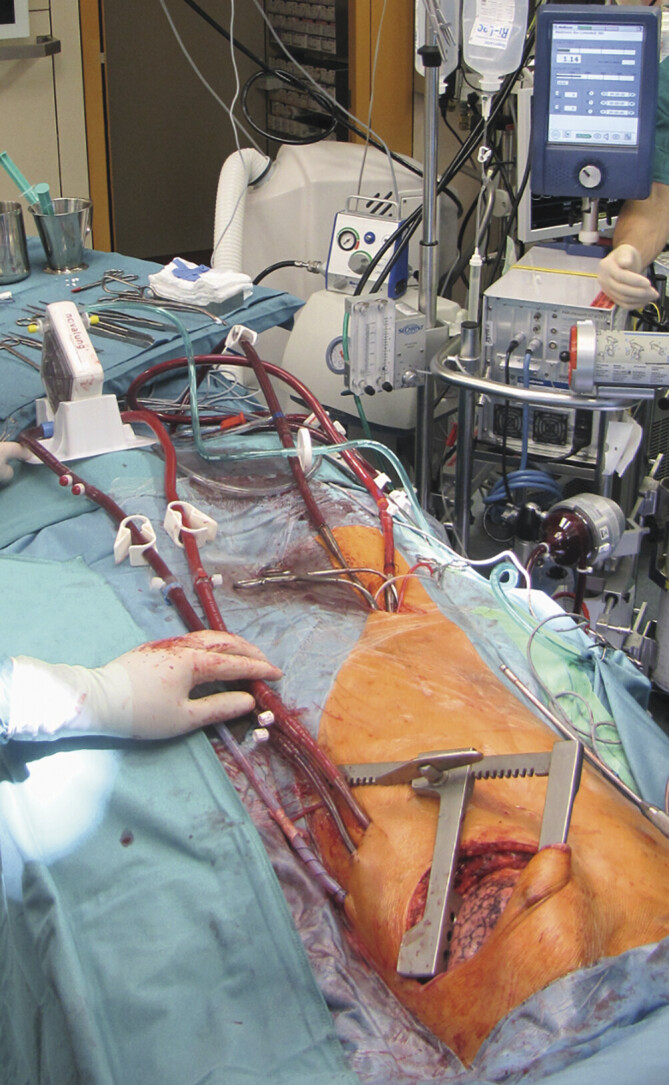
Etablierung eines zentralen PA-LA-Bridgings bei einer Patientin mit primärer pulmonaler Hypertension. Hierzu wird zunächst eine VA ECMO implantiert, anschließend die pumpenlose PA-LA-Novalung über eine Thorakotomie eingebracht und danach die VA ECMO wieder entfernt. Mit diesem Konzept können die Lungenstrombahn und der rechte Ventrikel entlastet werden. Die Patientin wurde anschließend wach bis zur Lungentransplantation geführt.

Aufgrund der Verfügbarkeit verschiedenster Kanülenarten und individueller Situationen ergibt sich eine Reihe weiterer, eher selten notwendiger Konfigurationsarten. Erwähnenswert ist z. B. das Erweitern durch zusätzliche venöse Drainagekanülen für eine venovenoarterielle (VV-A) oder venovenovenöse (VV-V) Konfiguration, um höhere Blutflüsse generieren zu können. Außerdem kann die Parallel- und Reihenschaltung zweier ECMO-Geräte erwogen werden, um etwa die Oxygenierungskapazität zu steigern oder um Kreislauf- und Lungensupport getrennt voneinander managen zu können 14 . Zur reinen Entlastung des rechten Herzens bei ausgeprägter pulmonaler Hypertension ist außerdem ein pumpenloser Shunt zwischen Pulmonalarterie und linkem Atrium über eine Novalung-Membran möglich (PA-LA; Abb. 3 ) 15 .

## ECMO-Management auf der Intensivstation

Die intensivmedizinische Betreuung von ECMO-Patient*innen erfordert eine enge und gut eingespielte interdisziplinäre Zusammenarbeit. Intensivmediziner*innen übernehmen dabei eine führende Rolle von der Patient*innenselektion und der Indikationsstellung über die ECMO-Anlage, das (Komplikations-)Management und die interdisziplinäre Koordination in den meisten Zentren 16 . Im Speziellen fallen Monitoring, Troubleshooting, Strategien zur Antikoagulation sowie die Bereitstellung mobiler ECMO-Programme für die auswärtige ECMO-Anlage bei instabilen Patient*innen in das Spektrum der behandelnden Intensivstationen.

### Monitoring

Wie auch bei allgemeinen intensivmedizinischen Patient*innen ist die regelmäßige klinische Inspektion und Untersuchung bei ECMO-Patient*innen unabdingbar, da Ischämien, Hyperämien oder Blutungen im Status häufig erkannt werden können. Dies beinhaltet auch die Inspektion des ECMO-Systems auf Thromben oder Ablagerungen. Neben den angeführten Maßnahmen zur Detektion einer differenziellen Hypoxie (Harlequin-Phänomen, s. o.) wird außerdem empfohlen, die Hämodynamik im Sinne der kardialen Funktion regelmäßig zu überprüfen. Dies gilt für jede Art der Konfiguration: Die Wahl des VV ECMO-Blutflusses sollte mit Bedachtnahme auf das Herzzeitvolumen getroffen werden und i. d. R. etwa 60–80% desselben betragen, da Abweichungen in beide Richtungen mit ineffizienter Oxygenierungsleistung einhergehen können 7 . Hierfür hat sich der Einsatz von Ultraschall im Allgemeinen und Echokardiografie im Speziellen bewährt und ist aus der klinischen Routine bei ECMO-Patient*innen nicht mehr wegzudenken 17 18 . Auch für die Evaluierung von Kanülenposition, Klappenfunktion und die Suche nach Komplikationen, wie z. B. Perikard- und Pleuraergüssen oder Blutungsquellen, ist der Ultraschall essenziell. Im VA ECMO-Setting sind zusätzlich Fragen nach Notwendigkeit und Positionierung von Deventing-Devices (z. B. Impella) und nach dem Weaning-Zeitpunkt sonografisch adressierbar 19 . Auch Methoden des kontinuierlichen hämodynamischen Monitorings werden mit Bedachtnahme auf ihre jeweiligen Limitationen empfohlen – insbesondere Bioreaktanz oder Ösophagus-Doppler-Sonden können unter laufender ECMO-Anwendung nur sehr bedingt zur Abschätzung der Hämodynamik herangezogen werden. Die Verwendung von Thermodilution oder Pulskonturanalyse sollte jedenfalls um echokardiografische VTI-Messungen (VTI: Velocity-Time-Index) im linksventrikulären Ausflusstrakt erweitert werden 20 . Laborkontrollen sollten ein- bis 2-mal täglich erfolgen und neben Standardlaborparametern auch Gerinnungsparameter mit Antikoagulationsmonitoring (s. u.), D-Dimer, Fibrinogen und Hämolyseparameter wie freies Hämoglobin, LDH und Bilirubin enthalten. Anzustrebende Hämoglobingrenzwerte werden mangels Evidenz für ECMO-Patient*innen nicht gesondert empfohlen und sollten sich nach dem individuellen Bedarf richten. An der ECMO selbst empfiehlt sich die Messung und regelmäßige Dokumentation von venösem Sog-, Transmembran- und Reperfusionsdruck sowie bei Vorhandensein auch von Echtzeitparametern wie Hämoglobin und venöser Prä-Oxygenator-Sauerstoffsättigung. Einmal täglich empfiehlt sich die Messung einer Post-Oxygenator-Blutgasanalyse mit einer FdO _2_ von 1,0, um anhand des Verlaufes inzipiente Oxygenatordysfunktionen zu erkennen.

### Troubleshooting

Blutungs- und thromboembolische Komplikationen gehören gemeinsam mit Infektionen zu den häufigsten potenziell lebensbedrohlichen Komplikationen. Auch kanülierungsassoziierte Probleme, kardiovaskuläre Komplikationen, technische Defekte und Oxygenatorversagen, Hämolyse, zusätzliches Organversagen, (Extremitäten-)Ischämien und Entgleisung von Metabolik oder Gerinnung sollten antizipiert werden. Für das entsprechende Troubleshooting ist ein funktionierendes interdisziplinäres Gesamtkonzept in enger Abstimmung mit Fachdisziplinen wie Chirurgie, Transfusionsmedizin und Radiologie notwendig. Auch ist darauf zu achten, dass alle direkt am Bett arbeitenden Berufsgruppen diese Probleme kennen und erkennen sowie auch adressieren können. Regelmäßige Schulungen und (Simulations-)Trainings sind dafür unabdingbar und stellen ein Qualitätsmerkmal von ECMO-Zentren dar.

### Antikoagulation

Trotz substanzieller Weiterentwicklung der ECMO-Systeme im Bereich der Biokompatibilität und Heparininnenbeschichtungen ist für den Betrieb extrakorporaler Gasaustauschverfahren i. d. R. eine systemische Antikoagulation notwendig. Allerdings fehlt die Evidenz für optimale Handlungsempfehlungen, und die Antikoagulationspraktiken variieren beträchtlich zwischen den Zentren. Die Gesellschaften empfehlen unfraktioniertes Heparin (UFH), Bivalirudin oder Argatroban 21 22 . Bei der Verwendung von UFH fanden wir in unserer eigenen Studie positive Auswirkungen von additivem Prostaglandin auf Blutungs- und Thromboserate 23 .

Grundsätzlich bestehen auch Möglichkeiten des therapeutischen Monitorings, z. B. mittels medikationsspezifischer Anti-Faktor-Xa-Assays (Anti-Xa; Zielwert i. d. R. 0,2–0,4 U/ml), aktivierter partieller Thromboplastinzeit (aPTT; Zielwert 1,5- bis 2,5-facher Ausgangswert), viskoelastischer Tests oder medikamentenspezifisch individualisierter Assays (z. B. Hemoclot). Allerdings ist diese Art, die Antikoagulationswirkung abzubilden, nach wie vor ungenau, handelt es sich doch um In-vitro-Tests, die weder die endotheliale Reaktion noch die komplexe Blut-Oberflächen-Interaktion mitberücksichtigen können. Außerdem unterliegen die Tests einer signifikanten Variabilität 21 .

In Situationen mit hohem Blutungsrisiko (z. B. Thrombopenie < 50 G/l) wird die systemische Antikoagulation als (relativ) kontraindiziert erachtet und üblicherweise pausiert. Bei komplexen hämorrhagischen oder thromboembolischen Komplikationen empfiehlt sich jedenfalls eine individuelle Strategie unter Einbeziehung von Spezialist*innen der Hämostaseologie. Insbesondere im frühen postoperativen Verlauf richtet sich die Antikoagulation nach der individuellen klinischen Einschätzung, wie etwa intraoperative Faktoren sowie Qualität und Menge der Drainageflüssigkeit, und daher einer Abwägung zwischen Thromboserisiko und Nachblutungsgefahr.

### ECMO-Retrieval

Die Stabilisierung mittels ECMO-Anlage in peripheren Krankenhäusern durch mobile Teams und der im Anschluss begleitete Transport wird als „ECMO-Retrieval“ bezeichnet. Dieses Konzept eignet sich für Patient*innen, die zum Zeitpunkt der telefonischen Vorstellung durch das auswärtige Krankenhaus hochgradig instabil, eingeschränkt transportfähig oder sich rapide verschlechternd darstellen und ein primärer Interhospitaltransfer ans Zentrum daher als zu riskant einzustufen ist. Da die Indikation zur Lungentransplantation häufig bei Patient*innen außerhalb des Zentrums gestellt wird, ermöglichen diese Programme einen Transfer auch bei hochgradig kompromittierter kardiorespiratorischer Situation 24 . Die Indikationsstellung erfolgt individuell und in Absprache mit den behandelnden Kolleg*innen.

## Indikationen

### Lungentransplantation

Verschiedenste Arten der extrakorporalen Zirkulation sind heute mehr denn je integraler Bestandteil von Lungentransplantationszentren. Die technischen Weiterentwicklungen der Membranen, Pumpen, Schlauchsysteme und Kanülen haben zum einen zur deutlichen Reduktion der ECMO-assoziierten Nebenwirkungen geführt (z. B. durch Zentrifugalpumpen, heparinbeschichtete Schlauchsysteme etc.). Des Weiteren sind extrakorporale Verfahren heute längst nicht mehr wenigen hochspezialisierten Zentren vorbehalten, sondern stehen relativ breit unterschiedlichsten Fachdisziplinen zur Verfügung. Gleichzeitig ist durch die geringere Invasivität die Nutzen-Risiko-Abwägung anders zu bewerten als vor einigen Jahren, weshalb sich auch die Anzahl der extrakorporalen Unterstützungen in der Lungentransplantation deutlich erhöht hat 1 . Besonders relevant sind 2 kürzlich erschienene Konsensusdokumente zur extrakorporalen Unterstützung im Kontext der Lungentransplantation. Sowohl durch die American Association for Thoracic Surgery (AATS) als auch durch die International Society for Heart & Lung Transplantation (ISHLT) wurden kürzlich Empfehlungen zum Einsatz von extrakorporalen Verfahren publiziert 25 26 .

### Präoperative ECMO

Präoperativ kommen ECMO-Verfahren als „Bridging“ zur Lungentransplantation zum Einsatz. In der Regel handelt es sich um Patient*innen auf der Warteliste, bei denen es zur raschen Verschlechterung der respiratorischen Funktion kommt oder in selektierten Fällen, um Patient*innen mit therapierefraktärem Lungenversagen ohne Aussicht auf Regeneration der Lunge, wie z. B. bei einigen Patient*innen mit COVID-19-ARDS 27 . Historisch war das Überleben der ECMO-gebridgten Patient*innen nach Lungentransplantation deutlich schlechter, weshalb sich ethische Fragestellungen in der Organallokation ergaben 28 . Allerdings kam es hier zur Trendwende, denn trotz sich ändernder Patientendemografie wird ein immer besseres Langzeitüberleben nach ECMO-Bridging erreicht 1 . Dies spiegelt sich auch in den publizierten Erfahrungen einzelner Zentren wider, die das 1-Jahres-Überleben in den Gesamtkohorten mit 69–100% angeben und in den rezenten Jahren eine stetige Verbesserung beobachten lassen 29 30 31 32 33 34 . Essenziell für den Erfolg des ECMO-Bridgings zur Lungentransplantation ist vor allem die Vermeidung einer Dekonditionierung. Idealerweise werden Patient*innen wach an der ECMO geführt und nehmen aktiv an der Physiotherapie teil. Eine femorale Kanülierung stellt keine Kontraindikation für eine Mobilisierung der Patient*innen dar und kann ohne zusätzliche Risiken erfolgen 35 . ECMO-Konfigurationen mit Doppellumenkanülen oder die „Sport“-VA-ECMO überzeugen zusätzlich durch die oftmals einfachere Handhabung während der Therapieeinheiten 36 . Wach gebridgte Patient*innen haben – vor allem bei längeren Wartezeiten an der ECMO – eine deutlich geringere postoperative Morbidität und schnellere Genesung, was sich etwa in einem kürzeren Intensiv- und Krankenhausaufenthalt widerspiegelt 37 38 . Die Indikation zur ECMO bei Patient*innen auf der Lungentransplantationswarteliste orientiert sich daher an der respiratorischen Erschöpfung trotz nicht invasiver Beatmung und hat ihren Stellenwert idealerweise noch vor Intubation und mechanischer Beatmung 39 40 .

Die ECMO-Konfiguration richtet sich nach der Grunderkrankung, dem aktuellen Zustand und der lokalen Gefäßsituation ( Abb. 1 ). Insbesondere bei längeren ECMO-Laufzeiten kann es zu einer Eskalation oder Deeskalation der ECMO-Konfiguration kommen. Bei Patient*innen mit zystischer Fibrose, chronisch obstruktiver Lungenerkrankung und der Mehrzahl der Patient*innen mit Lungenfibrose ist eine VV ECMO meist ausreichend. Aufgrund der Häufigkeit dieser Grunderkrankungen ist auch die VV ECMO die am häufigsten durchgeführte Bridging-Variante 29 31 32 33 40 41 . Allerdings kann es vor allem bei Patient*innen mit Lungenfibrose und ausgeprägter sekundärer pulmonaler Hypertension zur deutlichen Rechtsherzbelastung kommen, weshalb eine Erweiterung des Supports (wie oben beschrieben) notwendig werden kann. Bei Patient*innen mit primärer pulmonaler Hypertension ist das Rechtsherzversagen bei meist ausreichendem Gasaustausch führend. Hier sind die möglichst wache, periphere VA ECMO-Kanülierung oder in weiterer Folge eine Umkanülierung in PA-LA-Konfiguration die Modalitäten der Wahl. Eine frühzeitige ECMO-Anlage bei den ersten Anzeichen einer beginnenden Rechtsherzdekompensation wird in diesem Patient*innenkollektiv auch empfohlen, womit in erfahrenen Zentren sehr gute Langzeitergebnisse erzielt werden 25 42 .

### Intraoperative ECMO

Aufgrund der En-bloc-Implantationstechnik mit HLM während den Anfängen der Lungentransplantation war die HLM bis vor einigen Jahren ein noch immer verbreitetes Verfahren, obwohl sich die chirurgische Technik bereits zur sequenziellen bilateralen Lungentransplantation gewandelt hatte. Aufgrund der Invasivität der HLM und den damit assoziierten schlechteren Ergebnissen, ist die HLM heute nur noch komplexen Lungentransplantationen vorbehalten, wie etwa mit konkomitanten herzchirurgischen Eingriffen 43 44 .

**Abb. 4:**
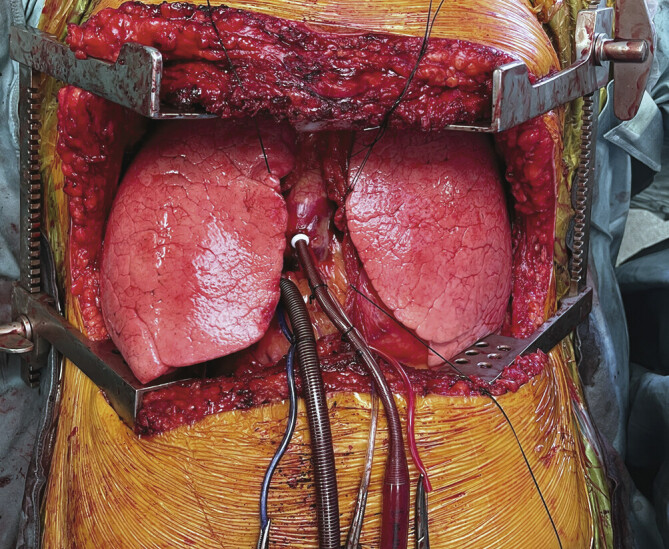
Intraoperativer Situs einer Lungentransplantation über eine Clamshell-Inzision, wobei eine zentrale VA Kanülierung auch über beidseitige Thorakotomien möglich ist.

Für die intraoperative ECMO gibt es einige absolute und relative Indikationen. Eine ausgeprägte primäre oder sekundäre pulmonale Hypertension, hämodynamische Instabilität, respiratorische Insuffizienz und chirurgisch-technische Gründe sind einige Beispiele, die eine ECMO notwendig machen können. Ermöglichung einer lungenprotektiven Beatmung, kontrollierte Reperfusion durch Entlastung des Lungenkreislaufes, bessere Exposition und eine bereits etablierte ECMO bei unerwarteten, chirurgisch-technischen Problemen sind weitere Gründe, die von verschiedenen Zentren unterschiedlich bewertet werden. Falls die Entscheidung zur ECMO getroffen wird, wird sowohl in den AATS- als auch ISHLT-Konsensusdokumenten die Verwendung einer VA ECMO-Konfiguration empfohlen; intraoperativ sollte der zentralen VA ECMO gegenüber der peripheren Kanülierung der Vorzug gegeben werden ( Abb. 4 ). Bei vorbestehender VV ECMO soll intraoperativ eine Umkonfiguration auf eine VA ECMO erwogen werden 25 26 . Die Frage, ob jede Lungentransplantation mit ECMO oder nur selektiv Patient*innen mit gewissen Risikofaktoren von Beginn an mit ECMO transplantiert werden sollen, bleibt auch in den Konsensusdokumenten offen. Wegen der sehr niedrigen Inzidenz der primären Graft-Dysfunktion (PGD) und besserem Langzeitüberleben ist die routinemäßige, zentrale VA ECMO über bilaterale, anteriore Thorakotomien an unserem Zentrum derzeit als Standard etabliert 45 46 .

### Postoperativ prolongierte ECMO

Nach erfolgter Transplantation können verschiedene Szenarien eine postoperative Prolongation der ECMO-Unterstützung notwendig machen. Bei Patient*innen mit primärer pulmonaler Hypertension ist aufgrund der Interaktion zwischen dem chronisch hypertrophen rechten Ventrikel, dem nun geringeren Widerstand des Gefäßbetts des Lungentransplantats und einer daraus resultierenden linksventrikulären diastolischen Dysfunktion ein progredientes Lungenödem zu erwarten. Daher werden beinahe alle Patient*innen mit primärer pulmonaler Hypertension mit einer prolongierten peripheren VA ECMO versorgt. Die VA ECMO erlaubt die Entlastung des Lungenkreislaufes und eine Remodellierung des linken Ventrikels, was i. d. R. innerhalb der 1. Woche erfolgt 47 48 49 50 .

Ein unzureichender Gasaustausch im Sinne einer PGD kann unabhängig von der zugrunde liegenden Diagnose nach jeder Transplantation auftreten. Die Einschätzung, welche Patient*innen von einer Prolongation der ECMO profitieren, kann u. U. schwierig sein. Meist ist der Trend von transösophagealer Echokardiografie, konsekutiven Blutgasanalysen und des pulmonalarteriellen Drucks ausschlaggebend. Eine frühzeitige Entscheidung zur ECMO-Prolongation ist jedoch vorteilhaft, um weitere Organschäden durch hohe Beatmungsinvasivität zu vermeiden 51 . Aufgrund der möglichen Komplikationen einer zentralen (Kontamination, Dislokation, Blutung) oder peripheren (Wundinfektion, Ischämie) prolongierten ECMO ist die Abwägung zur Prolongation sorgfältig zu treffen. Bei isolierter Gasaustauschstörung ohne hämodynamische Komponente wird aufgrund der niedrigeren Komplikationsrate im Vergleich zur VA ECMO eine VV ECMO empfohlen 25 .

## Weitere Indikationen

Neben der Lungentransplantation finden extrakorporale Gasaustauschverfahren bei verschiedensten thoraxchirurgischen Eingriffen Anwendung. Bei großen Mediastinaltumoren mit entsprechender Kompression der Trachea, des rechten Vorhofs oder der großen Gefäße kann es bei Narkoseeinleitung oder während der Präparation zur raschen Dekompensation kommen. Chirurgische Interventionen können bei diesen ausgedehnten Raumforderungen primär an der ECMO oder unter „ECMO-Stand-by“ sicher durchgeführt werden. Die ECMO-Konfiguration richtet sich wiederum nach der individuellen Situation 52 53 . Auch bei erweiterten Resektionen von fortgeschrittenen Tumoren unter Einbezug der V. cava inferior oder der Aorta descendens sowie der zentralen Atemwege finden verschiedene ECMO-Modalitäten Anwendung 54 55 .

Patient*innen mit eingeschränkter Lungenfunktion, sei es durch vorangegangene Resektionen oder durch akute oder chronische Lungenerkrankungen, können durch intraoperative ECMO-Unterstützung einem thoraxchirurgischen Eingriff unterzogen werden. Dies umfasst z. B. Patient*innen nach vorangegangener kontralateraler Pneumonektomie 56 57 , Lungenvolumenreduktion 58 oder septischen Eingriffen 59 . Auch bei offen chirurgischen oder endoskopischen Interventionen an den zentralen Atemwegen kann eine ECMO-Unterstützung erwogen werden 60 61 . Insbesondere bei intrathorakalen Tracheal- oder Carinaresektionen bietet die ECMO deutliche Vorteile. Durch ausreichend groß dimensionierte Kanülen kann mit einer VV ECMO ein vollständiger Lungenersatz erreicht werden. Die intermittierende Cross-Table-Beatmung wird somit überflüssig. Auch bei traumatischen Atemwegsverletzungen und konkomitantem Lungenversagen kann mithilfe der ECMO eine chirurgische Versorgung angestrebt werden 62 . Insgesamt kann die ECMO-Unterstützung diese herausfordernden Eingriffe wesentlich erleichtern oder sogar erst ermöglichen.

## Zusammenfassung

Extrakorporale Gasaustauschverfahren sind aus der modernen Thoraxchirurgie und insbesondere der Lungentransplantation nicht mehr wegzudenken. Durch den temporären Lungenersatz mittels ECMO können immer komplexere Patient*innen mit stetig besseren Ergebnissen behandelt werden. Trotz der steigenden Erfahrung in den letzten Jahrzehnten und zahlreicher Publikationen sind erst rezent Empfehlungen durch die internationalen Gesellschaften veröffentlicht worden. Zukünftig gilt es, die Komplikationsraten weiter zu senken, neue technische Entwicklungen kritisch zu prüfen und trotz verschiedenster Hürden qualitativ hochwertige Studien auf diesem Gebiet durchzuführen.
